# Assessment of pain in a Norwegian Emergency Department

**DOI:** 10.1186/s13049-015-0166-3

**Published:** 2015-10-29

**Authors:** Jostein Dale, Lars Petter Bjørnsen

**Affiliations:** Emergency Department, Clinic of Emergency Medicine and Prehospital Care, St. Olav’s Hospital, Trondheim, Norway

## Abstract

**Background:**

Although pain management is a fundamental aspect of care in emergency departments (EDs), inadequate treatment of pain is unfortunately common. There are multiple local protocols for pain assessment in the ED. This study evaluated whether the initial assessment and treatment of pain in the ED are in accordance with the in-hospital protocol of the ED at a Norwegian University Hospital.

**Materials and methods:**

Prospective data on pain assessment and initial treatment in the ED were collected from nursing and physician documentation. The patients’ perceptions of subjective pain were recorded using a numerical rating scale (NRS) that ranged from 0 to 10.

**Results:**

Seventy-seven percent of the 764 enrolled patients were evaluated for pain at arrival. Female patients had a higher probability of not being asked about pain, but there was no difference in the percentage of patients asked about pain with respect to age. Additionally, patients with low oxygen saturation and systolic blood pressure were less likely to be asked about pain. Of those with moderate and severe pain (58 %), only 14 % received pain relief.

**Discussion:**

Assessment and treatment of pain in the ED are inadequate and not in line with the local protocols. A focus on strategies to improve pain treatment in the ED is a necessary aspect of developing optimal acute patient care in Norway in the future.

## Introduction

Pain is often referred to as the fifth vital sign [1] and is one of the most common problems in patients who arrive at emergency departments (EDs) [2]. Patients usually expect that their pain will be addressed and treated [3]; however, despite patients’ expectations, there is suboptimal pain management in the ED. Pain is used as a quality indicator [3–5], and there are multiple guidelines for pain management [6, 7]. Acute pain may have adverse physiological and psychological effects [8, 9], but there is still inadequate treatment of pain in EDs [10, 11]. Of the patients who arrive at the ED with a painful condition, more than 50 % indicate that the pain is moderate to severe [12, 13]. Despite strategies to ensure safe and effective pain treatment in the ED, nurses rarely give analgesics before a physician sees the patient [14]. Previous studies have shown that even when nurses have local pain protocols, an unacceptable proportion of patients receive an inadequate assessment and pain treatment [15]. Health care workers in the ED often have limited knowledge of pain [16], and pain assessment is poorly documented in patient records [17]. Published guidelines for pain treatment recommend initial pain assessment of all ED patients [18], and if the patient indicates moderate to severe pain, treatment should be initiated [19]. Local ED protocol at St. Olav’s hospital was implemented in 2012 to ensure adequate pain assessment and treatment of all patients who arrive at the ED, but the performance in relation to the guidelines has not been evaluated. All patients should be asked to rate their subjective pain with a numerical rating scale (NRS) [20, 21], and patients with moderate to severe pain should be offered pain medications or appropriate intervention. Multiple studies have looked at assessment and management in the ED [22]. This prospective study evaluated whether the assessment and management of pain in a Norwegian ED was in line with the local protocols. A similar study has not previously been conducted in Norway.

## Materials and methods

In November 2012, a 20-day prospective study was conducted at St. Olav’s Hospital in Trondheim, Norway, where ED patient data were collected daily during the period of highest patient influx (noon-10 p.m.) [23]. Relevant quality indicators and data were chosen and included in the study design prior to collecting the data (including chief complaints, acuity level, vital signs, and pain score). Data was obtained from the regular nursing triage forms during or immediately after the initial assessment of the ED patients. All data were collected before the patient left the ED. Research assistants were constantly observing the patient care and activity in the ED while obtaining data immediately when available during the study period. The research assistants did not interfere with patient care or the nurses’ work, and there were no patient involvements. The physicians and nurses were aware of the ongoing study, but were not informed about what data that was obtained. St. Olav’s Hospital is a university hospital that serves approximately 280,000 local residents as a community hospital. In addition, the hospital has a regional function as a tertiary center for approximately 680,000 inhabitants. The ED primarily receives patients over 16 years of age and has an annual patient population of approximately 21,000 [23]. Data on all patients who arrived at the ED in the specified time period were manually recorded. Prospective data on the pain assessment and initial treatment in the ED were collected from nursing and physician documentation. The triage system, RETTS, is used to determine the acuity level (triage) in the ED (http://www.predicare.se) [24].

## Evaluation of pain

Patient perception of subjective pain should be evaluated at arrival according to local procedures at St. Olav’s Hospital. The pain is evaluated using a numeric rating scale (NRS), which measures the degree of pain on an 11-point scale from 0 to 10 [20, 21], where 0 indicates no pain and 10 indicates the worst imaginable pain. A score of 1–3 is defined as mild pain, 4–6 moderate pain, and 7–10 severe pain. The local protocol at St. Olav’s Hospital intends to ensure adequate pain assessment and treatment of all patients in the ED. The protocol is used for all adult patients who are awake (Glasgow Coma Scale ≥14) and do not have signs of threatened circulation or respiration (systolic blood pressure > 100 mmHg and SpO2 > 95 %). An ED nurse should evaluate all patients according to the NRS and then take action to relieve pain within 15 min. The treatment target is achievement of a NRS < 4 or patient pain tolerance.

## Statistical analysis

The statistics software SPSS version 22.0 (IBM, New York, USA) was used to analyze the data. The paired t-test was used for normally distributed data, and statistical significance was set at *p* < 0.05. The chi-square test was used to assess significant differences between the different patient groups. When more than 20 % of the squares in the chi-square calculation had an expected value less than 5, Fisher’s exact test was used. Unless otherwise specified, the percentage was calculated for the patients for whom the relevant variable was obtained.

## Ethics

The Regional Committee for Medical and Health Research Ethics (REK) in Norway and the Data Protection Officer at St. Olav’s Hospital, Trondheim, approved the study.

## Results

In the study, 764 patients were included (*n* = 764). The average age was 58.5 years (SD 26.7 years), and the proportion of women was 52.1 %. The patient distribution was characteristic of the general patient population distribution at our ED for all of 2012 with respect to age, gender, chief complaints (Emergency Symptoms and Signs (ESS) in RETTS), and acuity level. The proportion of patients who were asked about pain upon arrival at the ED was 77 % (*n* = 586), and the group was not significantly different from the group that was not asked about pain with respect to age, gender, and acuity level. In the group that was not asked about pain (23 %, *n* = 178), 10.5 % of the patients had a systolic blood pressure below 100 mmHg (average 132 mmHg, SD 26 mmHg), whereas in the group that was asked about pain, 2.6 % (average 137 mmHg, SD 23 mmHg) had a lower blood pressure (*p* = 0.000). There was also a significant difference in the average blood pressure between the two groups (*p* = 0.022). At the same time, significantly more patients with oxygen saturation below 95 % were not asked about pain (25.8 % versus 12.0 %, χ^2^ = 15.772, df = 1, *p* = 0,000). The difference in the mean oxygen saturation was relatively small but significant, with values of 97.5 % and 95.8 %, respectively, among the patients asked about pain and those not asked about pain (*p* = 0.011). The most common chief complaints in both groups were abdominal pain, chest pain, breathing difficulties, and neurological and infectious conditions. The differences between the patients who were and were not asked about pain are shown in Table 1.

**Table 1:** Comparison of patient groups according to the pain assessment

	Pain assessment (*n* = 586)	No pain assessment (*n* = 178)
Age (Mean ± SD)	57.9 ± 21.5	60.5 ± 22.3
Female	49.9 %	59.4 %*
Systolic BP < 100 mmHg	2.6 % (*n* = 15)	10.5 % (*n* = 13)*
O_2_ Sat < 95 %	12.0 % (*n* = 70)	25.8 % (*n* = 32)*
Age > 64 years	44.6 % (*n* = 261)	50.3 % (*n* = 88)
Triage level (RETTS):		
Red	9.6 %	8.4 %
Orange	29.4 %	28.1 %
Yellow	46.3 %	52.2 %
Green	14.4 %	10.7 %
Blue	0.3 %	0.6 %

*Statistically significant, *p* < 0.05

**Fig. 1:**
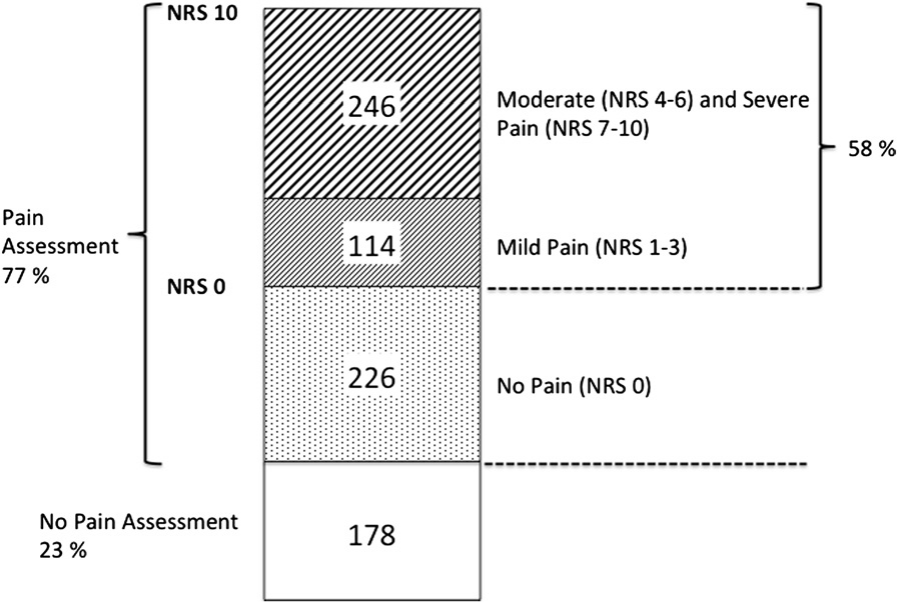
Overview of pain assessment in the ED

Of the patients who were asked about pain (*n* = 586), 58 % (*n* = 340) stated that they were in pain (Fig. 1) and 66.5 % (*n* = 226) indicated that they had pain that was more severe than mild (NRS > 3). There was no significant age difference between the patients who were asked about pain and those who were not asked about pain; however, there were significantly more women than men who were not asked about pain (80.5 and 73.8 %, respectively; χ^2^ = 4.733, df = 1, *p* = 0.03).

According to the local ED procedures, the goal is to treat all patients with moderate and severe pain. The average pain scores in patients who reported pain on arrival to the ED was 4.9 (median 5.0). There was no significant difference in the pain scores between those who were and were not treated for their pain (4.59 versus 4.91). Of the group of patients with moderate and severe pain (NRS > 3; n = 226), 14.2 % (*n* = 32) were treated with pain medications. There was no difference in age (p = 0.544) or gender (*p* = 0.188) between the patients who did or did not receive analgesia. The study was not designed to identify the drugs or interventions that were used for pain management.

## Discussion

This study reveals a known phenomenon; specifically, patients do not receive optimal assessment and treatment of pain in the ED. Twenty-three percent of patients were not asked about their pain, and only 14.3 % of the patients who reported moderate to severe pain received treatment for their pain. A study by Barletta and colleagues showed a significant difference in the pain scores between patients who received pain treatment and those who did not [25], but there was no such trend in our study. Additionally, the average pain score of 4.9 is somewhat lower than that found in other studies [26].

Other hospitals that also have guidelines for pain management additionally struggle with low compliance with pain level documentation. Only 49.2 % of the patients were asked about their pain in triage [27], and, of these patients, 41.3 % had severe pain, 38.8 % had moderate pain, and 19.8 % had mild pain. Although pain management is a fundamental aspect of emergency care, there are several attitudinal and structural barriers to efficient and adequate pain management in the ED [28]. Interestingly, some of the nurses had written explanations for the lack of assessment of pain or treatment on the patient assessment form. These reasons included communication barriers and language problems, altered mental status and intoxication, dementia or difficulty in describing pain, intermittent or occasional nature of the pain, patient received prehospital treatment or analgesia at home, and patient refusal. It is worth noticing that there are significant inter-rater differences in pain score on ED arrival between patients and emergency health care providers [29], and nurses tend to underestimate pain often than physicians [30, 31]. It is known that the subjective experience of pain, provider gender, misapprehension, prejudice among health care providers, stress, work pressure, and relatively short and intense patient interaction in the ED may affect the quality of pain management [32–38]. Additionally, a patient’s age, gender, race, and ethnicity may be of significance [39, 40]. Young age and female gender are factors that increase the likelihood of pain [26, 41], but we found no difference between the gender and age of patients who experienced pain. However, we found that female patients were less likely to be assessed for pain. Emergency signs and symptoms (ESS) coincided with previous studies in our ED [42], and patients who were not asked about pain presented with similar issues and priorities compared with the group that was asked about pain.

Pain in the elderly is a common problem in the ED [43], and pain management in the elderly can be challenging because these patients are at an increased risk for adverse effects of analgesics [44]. Older patients are less likely to experience pain in the ED, and increasing age predicts inadequate analgesia administration [45]. Nevertheless, we found no significant difference in the proportion of patients over 64 years of age who were and were not asked about pain.

Of the patients with moderate to severe pain, few received pain management in the ED. There was no difference in age or gender between the patients who did or did not receive analgesics. In one study [46], approximately 50 % of the patients were asked about pain, which is lower than the percentage in our study. Forty-three percent stated that they had moderate to severe pain, but only 25 % of these patients received pain medication. In comparison, only 14.3 % of similar patients in our ED received any treatment for pain. Other studies reveal that 50–60 % of patients who report pain in the ED receive pain management [26].

Adequate pain management should be a primary goal of health care professionals in the ED, but the pain assessment and treatment performed by nurses have been shown to be suboptimal [36]. The documentation of pain during triage and protocols that give nurses the opportunity to initiate treatment are associated with better pain management [27]. However, guidelines and protocols have little effect on the improvement of pain treatment as a single initiative [47]. Current local protocol does not require pain assessment in all patients in our ED. A series of interventions intended to improve pain treatment in the ED has been suggested, but there is no currently accepted universal model [48]. Other countries have accepted pain management in the ED as a quality indicator [49] and this will likely be adapted in Norway [50]. The first step in improving the pain assessment and management in the ED is to accurately and systematically assess every patient. To improve the assessment and treatment of pain in patients in the ED, a local framework focusing on knowledge, communication, emergency organization, and patient flow must be established [51, 52]. This approach requires a change in attitude among health workers and frequent evaluation and feedback [47, 53]. The ED staff needs to have knowledge of the patient population and to continuously measure the quality of pain management. Adapting a local protocol alone seems to give suboptimal pain assessment in the ED.

## Conclusion

Pain is one of the most common reasons for seeking emergency medical care; thus, it is important for healthcare professionals to focus on effectively assessing and treating pain. Despite guidelines and in-service educational programs, the assessment and treatment of pain in our ED are inadequate. This prospective study reveals that the management of pain is not in accordance with internal procedures. Pain management is accepted as a quality indicator of care, and additional focus on strategies to improve pain management in the ED is necessary to ensure that all patients receive optimal pain assessment and treatment.
